# Alpha‐synuclein co‐pathology in a real‐world early Alzheimer's disease cohort

**DOI:** 10.1002/alz.71240

**Published:** 2026-03-24

**Authors:** Tamara Shiner, Talya Nathan, Mori Hai Levy, Aya Bar David, Nurit Omer, Anan Abu Awad, Elissa Ash, Mali Gana Weisz, Orly Goldstein, Yifat Alcalay, Keren Regev, Jennifer Lamoureux, Kendall Van Keuren‐Jensen, Cornelis Blauwendraat, Roy N Alcalay, Noa Bregman

**Affiliations:** ^1^ Cognitive Neurology Unit, Neurological Institute Tel Aviv Medical Center Tel Aviv Israel; ^2^ Gray Faculty of Medical & Health Sciences Tel Aviv University Tel Aviv Israel; ^3^ Sagol School of Neuroscience Tel Aviv University Tel Aviv Israel; ^4^ Movement Disorders Unit, Neurological Institute Tel Aviv Medical Center Tel Aviv Israel; ^5^ Laboratory of Biomarkers and Genomics of Neurodegeneration Tel Aviv Israel; ^6^ Division of Clinical Laboratories Tel Aviv Medical Center Tel Aviv Israel; ^7^ Neuroimmunology Unit Tel Aviv Medical Center Tel Aviv Israel; ^8^ Amprion, Inc. San Diego California USA; ^9^ Center for Alzheimer's and Related Dementias National Institute on Aging and National Institute of Neurological Disorders and Stroke National Institutes of Health Bethesda Maryland USA; ^10^ Bioinnovation and Genome Sciences The Translational Genomics Research Institute Phoenix Arizona USA

**Keywords:** α‐synuclein, α‐synuclein seed amplification assay, Alzheimer's disease, anti‐amyloid therapy, cerebrospinal fluid biomarkers, co‐pathology, disease heterogeneity, disease‐modifying therapy, early Alzheimer's disease, Lewy body pathology, olfactory impairment, rapid eye movement sleep behavior disorder, real‐world cohort

## Abstract

**BACKGROUND:**

Most Alzheimer's disease (AD) cases show mixed pathology, with α‐synuclein (αSyn) aggregates present in a substantial proportion. The cerebrospinal fluid (CSF) α‐synuclein seed amplification assay (αS‐SAA) enables in vivo detection of pathogenic αSyn aggregates, but its clinical significance remains unclear.

**METHODS:**

We prospectively evaluated 108 individuals with mild cognitive impairment or mild dementia due to suspected AD undergoing lumbar puncture for anti‐amyloid therapy (ATT) eligibility. CSF AD biomarkers and αS‐SAA were analyzed alongside cognitive, olfactory, and rapid eye movement sleep behavior disorder (RBD) assessments.

**RESULTS:**

Of 65 participants with biomarker‐confirmed AD, 21 (32.3%) were αS‐SAA positive. Positivity was linked to older age at testing and self‐reported olfactory impairment (*P* = 0.004), but not other demographic or cognitive features. Within the αS‐SAA–positive group, RBD presence correlated with faster seeding kinetics.

**CONCLUSIONS:**

αS‐SAA positivity is common in early AD and associated with olfactory dysfunction. Longitudinal follow‐up is required to test if assay status predicts response to ATTs.

## Highlights

- α‐synuclein (αS) co‐pathology was detected in 32% of early Alzheimer's disease (AD) cases.
- α‐synuclein seed amplification assay (αS‐SAA) positivity was associated with self‐reported olfactory impairment.
- Rapid eye movement sleep behavior disorder was linked to faster αs seeding kinetics.
- Findings support biological heterogeneity in early biomarker‐confirmed AD.
- αS‐SAA may inform patient stratification for amyloid‐targeting therapies.

## 1 INTRODUCTION

Alzheimer's disease (AD) remains the most common cause of dementia in older adults and is characterized by considerable clinical and pathological heterogeneity.^1^ This heterogeneity is critically important for predicting outcomes in trials of disease‐modifying therapies.^2^ AD is defined by a specific etiology, marked by characteristic AD neuropathological changes (ADNCs), which include amyloid beta (Aβ) plaques, neurofibrillary tangles (NFTs), and the Consortium to Establish a Registry for Alzheimer's Disease (CERAD) neuritic plaque score, which reflects the density of neuritic plaques in key neocortical regions.^3^, ^4^, ^5^ Lewy bodies are the pathological hallmark of Lewy body diseases, including Parkinson's disease (PD); Parkinson's disease dementia (PDD); and dementia with Lewy Bodies (DLB), the second most common neurodegenerative dementia after AD.^6^, ^7^

The neuropathological changes in AD are often accompanied by coexisting proteinopathies including alpha synuclein (αSyn) and TAR DNA‐binding protein 43 (TDP‐43) pathology as shown in *post mortem* evaluations. Approximately one third of clinically diagnosed AD cases exhibit concomitant Lewy body pathology on autopsy,^8^ and up to two thirds of patients with cognitive impairment aged 80 to 89 have significant mixed pathology.^9^ Cerebrospinal fluid (CSF) misfolded αSyn aggregates (αSyn‐seeds) are an accepted biomarker for synucleinopathies such as PD and DLB.^10^ These aggregates can be detected in CSF using the α‐synuclein seed amplification assay (αS‐SAA), an in vitro technique that enables identification of αSyn pathology in vivo.^11^

The effect of αSyn pathology in AD is still being investigated. The presence of αSyn pathology has been reported to exacerbate cerebral hypometabolism and cognitive decline in AD;^12^ however, this study was performed in the Alzheimer's Disease Neuroimaging Initiative (ADNI), which despite being a well‐characterized, multicenter cohort does not expressly screen and exclude for patients with symptoms in keeping with synucleinopathies and patients across the Lewy body dementia spectrum.^13^ Two recent studies using the αS‐SAA in AD cohorts spanning mild cognitive impairment (MCI) through mild dementia found αS‐SAA positivity in ≈ 30% of cases, a finding linked to more rapid clinical decline.^14^, ^15^

Here we aimed to examine the prevalence and potential impact of αS‐SAA positivity in individuals diagnosed with biomarker‐confirmed early AD, in a real‐world cohort undergoing assessment for amyloid‐targeting therapy (ATT) eligibility. We further investigated whether specific clinical features could predict αS‐SAA positivity in this early AD population.

## 2 METHODS

### 2.1 Study population

The study included individuals who underwent a lumbar puncture (LP) for MCI or mild dementia due to suspected AD, prior to consideration of suitability for ATTs. All individuals who underwent an LP between May 2024 and February 2025 at the Tel Aviv Medical Center (TLVMC) day‐admission unit were invited to participate and were recruited consecutively. All participants provided written informed consent, and the procedures were approved by the institutional Helsinki ethics committee (0613‐22‐TLV).

Demographic data and medication history were collected and participants underwent testing with the Mini‐Mental State Examination (MMSE).^16^ They also completed questionnaires including the REM Sleep Behavior Disorder Screening Questionnaire (RBDSQ)^17^ and the Lawton Instrumental Activities of Daily Living (IADL)^18^ scale, the Depression Anxiety Stress Scale (DASS),^19^ and the Geriatric Depression Scale (GDS).^20^ Subjective olfactory function was assessed via direct questioning regarding perceived reduction or alteration in the sense of smell. In accordance with established criteria, RBD was defined as an RBDSQ score of ≥ 5.^21^ All participants underwent brain magnetic resonance imaging (MRI) prior to LP to exclude significant structural or vascular pathology that could account for cognitive impairment.^1^

**RESEARCH IN CONTEXT**

1. **Systematic review**: The authors reviewed published studies on α‐synuclein co‐pathology in Alzheimer's disease (AD) using PubMed and recent conference abstracts. Prior neuropathological and α‐synuclein seed amplification assay (αS‐SAA) studies have demonstrated α‐synuclein aggregates in approximately one third of AD cases, but data from real‐world, biomarker‐confirmed early AD cohorts remain limited.
2. **Interpretation**: Our findings show that αS‐SAA positivity is common in early AD and associated with olfactory impairment and rapid eye movement sleep behavior disorder features. These results extend previous work by confirming α‐synuclein co‐pathology in a consecutively recruited, clinically defined population evaluated for amyloid‐targeting therapy eligibility.
3. **Future directions**: Future studies should determine whether αS‐SAA status predicts differential progression or treatment response to amyloid‐targeting therapies. Longitudinal and multicenter studies incorporating imaging and blood‐based biomarkers will be needed to fully clarify the clinical impact and utility of α‐synuclein biomarkers in early AD.

### 2.2 CSF collection and analysis

LP was performed according to international guidelines.^22^ CSF biomarkers, including total tau (t‐tau), hyperphosphorylated tau (p‐tau181), and amyloid beta 42 (Aβ42), were analyzed using the Euroimmun immunoassay (EUROIMMUN AG), based on enzyme‐linked immunosorbent assay (ELISA) technology and standardized protocols. Biomarkers results were considered indicative of AD pathology when p‐tau181 levels were > 61 pg/ml, Aβ42 levels were < 570 pg/ml (borderline: 570–630 pg/ml), and t‐tau levels were > 452 pg/ml (borderline: 290–452 pg/ml).^23^

### 2.3 SAAmplify αSyn (CSF)

The αS‐SAA was performed in the Amprion Clinical Laboratory (CLIA ID No. 05D2209417; CAP No. 8168002) using a method validated for clinical use in accordance with Clinical Laboratory Improvement Amendment (CLIA) requirements. Each sample was analyzed in triplicate in a 96‐well plate using a reaction mixture comprised of 100 mM PIPES pH 6.5, 0.44 M NaCl, 0.1% sarkosyl, 10 µM ThT, 0.3 mg/mL recombinant αSyn, and 40 µL CSF, in a final volume of 100 µL. Two borosilicate glass beads were included in each well, and positive and negative assay quality control samples were included on each plate. Plates were sealed with optical adhesive film, placed into the chamber of a BMG LABTECH FLUOstar Ω Microplate Reader, and incubated at 42°C with cycles of 1 minute of shaking followed by 14 minutes of rest with fluorescence measured after every shaking cycle (excitation wavelength 440 nm, emission 490 nm). After a total incubation time of 20 hours, the maximum fluorescence for each well was determined and an algorithm applied to the triplicate determinations for each sample for result classification.

### 2.4 Statistical analysis

Normality and homogeneity of variance were explored using Q‐Q plot and the Levene homogeneity test. Demographic and clinical characteristics of the patients were analyzed using a two‐tailed Student *t* test or chi‐squared test. Within the group of αS‐SAA–positive participants, those with RBD versus no RBD were compared using an analysis of covariance (ANCOVA) with RBD (yes or no) as between‐subject factors and the time to threshold (TTT) and time to maximum slope as dependent variables, with age at testing, sex, and CSF protein levels as covariates. Eta squared served to estimate effect size.

A chi‐square test of independence was performed to examine the association between self‐reported olfactory loss (yes/no) and αS‐SAA results (positive/negative).

All analyses were conducted with Statistical Package for the Social Sciences (SPSS) software (version 29; SPSS, Inc.), and the alpha level was set at 0.05.

## 3 RESULTS

CSF samples from 108 individuals who underwent diagnostic LP were analyzed. After excluding those without an AD‐consistent CSF biomarker profile (*n* = 40) and those with inconclusive αS‐SAA outcomes (*n* = 3), 65 participants remained with biomarker‐confirmed AD and definitive αS‐SAA outcomes. Among these, 21 (32.3%) were αS‐SAA positive.

There were no significant differences in demographics, clinical characteristics, or AD‐related CSF biomarkers between αS‐SAA positive (+) and αS‐SAA negative (−) (Table 1) participants except for age at LP, which was higher among αS‐SAA+ participants (74.71 years vs. 71.25 years, *P* = 0.050). MMSE and questionnaire scores did not differ significantly between groups.

**TABLE 1:** Characteristics of participants by αS‐SAA status.

	All subjects	αS‐SAA −	αS‐SAA +	*P* value
Total number of participants	65	44 (67.69%)	21 (32.3%)	
Age at testing (years) [SD]	72.37 [6.68]	71.25 [6.96]	74.71 [5.46]	**0.050** ^§^, ^*^
AAO (years) [SD]	70 [6.77]	68.92 [7.26]	71.95 [5.43]	0.109^§^
Disease duration (years) (*n* = 56)	2.63 [1.65]	2.69 [1.56]	2.50 [1.85]	0.678^§^
Sex, % female [SD]	32 [18.2]	24 (54.5%)	8 (38.7%)	0.215^§§^
Education (years) [SD] (*n* = 60)	14.93 [3.91]	15.25[4.35]	14.25 [2.82]	0.344^§^
*APOE* ε4 positive N (%) (*n* = 44)	18 (41%)	13 (43.3%)	5 (35.7%)	0.632^§§^
MMSE score [SD] (*n* = 62)	23.47 [4.37]	22.95 [4.82]	24.55 [3.03]	0.11^§^
IADL score [SD] (*n* = 58)	6.29 [1.56]	6.41 [1.53]	6.1 [1.64]	0.483^§^
DASS depression score [SD] (*n* = 52)	4.58 [4.49]	4.27 [4.25]	5.11 [4.96]	0.544^§^
DASS Anxiety score [SD] (*n* = 52)	3.44 [3.37]	3.15 [3.54]	3.95 [30.8]	0.40^§^
DASS Stress score [SD] (*n* = 51)	5.51 [5.33]	5.69 [5.91]	5.21 [4.31]	0.73^§^
RBDQ average mark [SD] (*n* = 56)	2.46 [2.38]	2.16 [2.37]	3.05 [2.34]	0.18^§^
RBD yes (%) (*n* = 56)	8 (14.28%)	4 (10.81%)	4 (21.05%)	0.30^§§^
Decreased smell Y (%) (*n* = 57)	8 (14.03%)	2 (5.12%)	6 (33.33%)	0.004^§§^, ^*^
CSF t‐protein [SD] (pg/ml)	50.48 [19.41]	48.72 [19.33]	54.03 [19.57]	0.31^§^
CSF t‐tau (pg/ml) [SD]	555.65 [250.71]	564.41 [256.73]	537.29 [242.73]	0.68^§^
CSF p‐tau (pg/ml) [SD]	135.17 [91.37]	127.05 [43.88]	152.19 [148.82]	0.45^§^
CSF Aß_42_ (pg/ml) [SD]	370.88 [140.95]	379.08 [146.82]	353.71 [1.95]	0.48^§^
Ratio amyloid/ptau [SD]	3.35 [1.69]	3.31 [1.58]	3.43 [1.95]	0.81^§^
TTT (1000 RFU) [h]_Med [SD]			9.71 [1.66]	
Fmax [RFU]_Med [SD]			51065.85 [11405.75]	
AUC‐Fluoro_Med [SD]			1490090361.02190500000000 [539682894.422617200000000]	
Max Slope_Med [SD]			12.18 [4.3]	
Time to Max Slope [h]_med [SD]			10.30 [1.68]	

Abbreviations: αS‐SAA −/+, alpha‐synuclein seed amplification assay negative or positive; AAO, age at onset; AD, Alzheimer's disease; *APOE*, apolipoprotein E; Aß, amyloid beta; AUC, area under the curve; CSF, cerebrospinal fluid; DASS, Depression Anxiety Stress Scales; Fmax, maximum fluorescence; IADL, Instrumental Activities of Daily Living; Med, median; MMSE, Mini‐Mental State Examination; p‐tau, hyperphosphorylated tau; RBDQ, REM Sleep Behavior Disorder Questionnaire; RBD, rapid eye movement sleep behavior disorder; RFU, relative fluorescence units; SD, standard deviation; t‐protein, total protein; t‐tau, total tau; TTT, time to threshold; YOE, years of education.

^§^ Independent sample *t* test equal variances,

^§§^Pearson chi‐square,

^*^ *p* ≤ 0.05.

A chi‐squared test of independence was conducted to assess the association between self‐reported olfactory impairment and αS‐SAA status. Among the 57 participants with available olfactory data, 8 reported reduced smell perception. Of these, 6 were αS‐SAA+, compared to 2 of 49 participants without olfactory loss. This association was statistically significant (*χ* ^2^[1, *N* = 57] = 8.12, *P* = 0.004).

### 3.1 Kinetic analysis and baseline clinical parameters

**FIGURE 1:**
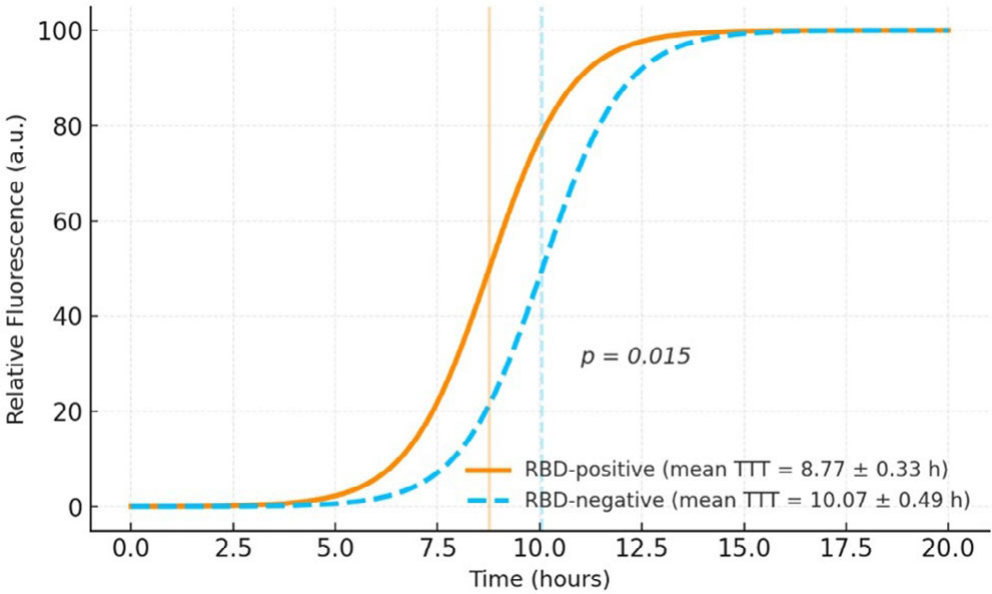
FIGURE 1 αS‐SAA seeding kinetics by RBD status. Vertical translucent lines mark each group's mean TTT ± standard error of the mean. αS‐SAA, alpha‐synuclein seed amplification assay; RBD, rapid eye movement sleep behavior disorder; TTT, time to threshold.

Exploratory analyses within the αS‐SAA+ group revealed that after controlling for age at testing, sex, and CSF total protein levels (given their potential influence on αS‐SAA outcomes^24^), patients with RBD (*n* = 4) exhibited a significantly faster median TTT compared to those without RBD (*n* = 15; mean TTT: 8.76 vs. 10.06; *F*[1,15] = 8.42, *P* = 0.012, *η* ^2^ = 0.376; ANCOVA). Similarly, time to maximum slope was shorter in patients with RBD compared to those without RBD (mean: 9.35 vs. 10.64; *F*[1,15] = 7.76, *P* = 0.015, *η* ^2^ = 0.357; Figure 1). Both associations remained significant irrespective of whether total protein was included as a covariate. No additional associations were observed with baseline demographic, cognitive, or functional measures.

## 4 DISCUSSION

Approximately one third (32%) of individuals with biomarker‐confirmed early AD undergoing evaluation for ATTs tested positive for CSF αSyn aggregates using the SAA. This finding aligns with prior pathological and clinical studies,^8^, ^12^, ^14^, ^15^ but importantly, our data derive from a real‐world cohort of early‐stage, clinically defined AD (MCI or mild dementia), with explicit exclusion of DLB and PDD. These results support the concept that αSyn co‐pathology may contribute meaningfully to the biological heterogeneity of early AD.^25^ It is noteworthy that in sporadic AD, αSyn pathology is most prominent within the amygdala,^26^ and that αS‐SAA demonstrates reduced sensitivity for detecting amygdala‐predominant αSyn;^27^ therefore, the rate of αSyn positivity by seeding assay in early AD is likely to be an underestimation, in keeping with the findings of higher rates of αSyn pathology on *post mortem* evaluation.^28^

We also identified a significant association between self‐reported olfactory impairment and αS‐SAA positivity. This is despite none of the patients having clinically suspected synucleinopathies based on specialist neurological assessment. Previous studies have reported distinct cognitive trajectories among AD patients with positive αS‐SAA results. Specifically, αS‐SAA–positive individuals demonstrated greater visuospatial impairment at baseline^14^ and more pronounced cognitive decline during follow‐up.^12^, ^14^, ^29^, ^30^ Moreover, in patients with established synucleinopathies such as DLB or PDD, coexisting AD pathology has been associated with a shorter interval between motor symptom onset and dementia, as well as poorer survival.^31^ Therefore, identifying a readily measurable clinical correlate of αS‐SAA positivity may support improved patient stratification, even in the absence of specialized biomarker assays that are not yet widely available in clinical practice.

We also found that participants with self‐reported RBD symptoms on a structured questionnaire exhibited faster seeding activity than those without, as reflected by a shorter TTT and faster time to maximum slope on αS‐SAA kinetics. RBD is a core clinical feature of DLB,^32^ a disorder characterized by rapid progression and overlapping αSyn and AD pathologies. Furthermore, RBD has been linked to an increased risk of dementia in patients with PD.^33^ It has previously been shown in patients with PD^34^ and DLB^35^ that more prominent αSyn seeding kinetic profiles are associated with more rapid cognitive decline. Similarly, in AD, shorter TTT values have been correlated with greater clinical deterioration.^14^ Although such differences in baseline characteristics were not observed in our cohort, the association between faster seeding activity and RBD symptoms in patients with AD raises the possibility of a more aggressive disease course among αSyn–positive cases. However, this was an exploratory analysis based on a small sample, and confirmation in larger, longitudinal cohorts is warranted.

One of the main strengths of this study is that it uses data from a real‐world clinical setting and consecutively recruited individuals, with biomarker‐confirmed early AD enhancing generalizability beyond highly selected research cohorts. Importantly, none of the included patients had a clinical suspicion of DLB or PDD, strengthening the interpretation that these findings reflect true αSyn co‐pathology within early AD rather than misdiagnosed primary synucleinopathies. This raises the possibility that αSyn co‐pathology in AD may be more prevalent in real‐world clinical settings than previously recognized.

This study has several limitations. Although all participants were clinically diagnosed with probable AD and did not fulfill diagnostic criteria for DLB, motor symptoms were not systematically assessed. Brain MRI was available for a subset of participants as part of routine clinical care and was used primarily to exclude significant structural or vascular pathology; systematic collection and harmonization of MRI data has now been initiated to enable future quantitative regional atrophy analyses. Apolipoprotein E genotype data were unavailable for approximately one third of participants, limiting a more comprehensive analysis of its potential interaction with αSyn co‐pathology. Furthermore, longitudinal follow‐up data were not yet available to evaluate whether αS‐SAA positivity correlates with disease progression or treatment response among those who initiated ATT (*n* = 31).

Disease‐modifying therapy (DMT) for AD is defined as an intervention that produces enduring changes in the clinical progression of AD by altering the underlying pathophysiological mechanisms of the disease that lead to neuronal loss.^36^ Over the past two decades, therapeutic development efforts have focused on modulating core disease processes, including Aβ42 deposition, tau pathology, neuroinflammation, and metabolic dysfunction.^37^ With the approval of ATTs aimed at slowing disease progression in early‐stage AD, the observed variability in treatment response and the occurrence of adverse events highlight the need for a more comprehensive understanding of patient‐specific biological factors. Incorporating αSyn biomarkers such as the αS‐SAA into early AD cohorts may enhance patient stratification and help elucidate the sources of treatment variability in clinical trials.

## CONSENT STATEMENT

All participants provided written informed consent.

## CONFLICT OF INTEREST STATEMENT

The authors declare no conflicts of interest. J.L. is an employee of Amprion.

## FUNDING INFORMATION

No funding was received for this research.

## Supporting information



Supporting Information
